# Removal of an embedded gastric fishbone by traction-assisted endoscopic full-thickness resection

**DOI:** 10.1055/a-2268-5934

**Published:** 2024-03-08

**Authors:** Shuzhen Chen, Songsong Ying, Cailian Xian, Yongqiang Li, Wenyan Jiang

**Affiliations:** 1 74668 Department of Gastroenterology and Hepatology, Guangzhou Digestive Disease Center, Guangzhou First People's Hospital, School of Medicine, South China University of Technology, Guangzhou, China; 2 74668 Department of Ultrasound, Guangzhou First Peopleʼs Hospital, School of Medicine, South China University of Technology, Guangzhou, China

A 65-year-old man was referred to our hospital with a half-year history of upper abdominal pain. Endoscopy showed a submucosal eminence on the anterior wall of the gastric antrum ( Fig. 1 **a** ). Endoscopic ultrasonography (EUS) revealed a hyperechoic lesion in the gastric submucosa ( Fig. 2 ). A computed tomography (CT) scan showed a long, high density shadow in the gastric antrum, locally protruding into the serosal cavity ( Fig. 3 ). Emergency endoscopy was performed with the patient under general anesthesia and with endotracheal intubation ( Video 1 ).

**Fig. 1:**
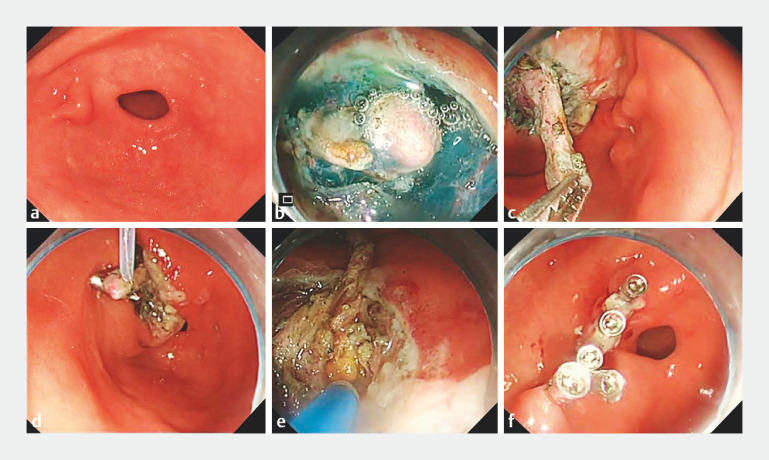
Endoscopic images showing: **a** a submucosal eminence on the anterior wall of the gastric antrum; **b** partial exposure of the fishbone; **c** attempts to extract the fishbone using foreign body forceps; **d** snare traction being employed; **e** endoscopic full-thickness resection being performed; **f** closure of the perforation with metal clips.

**Fig. 2:**
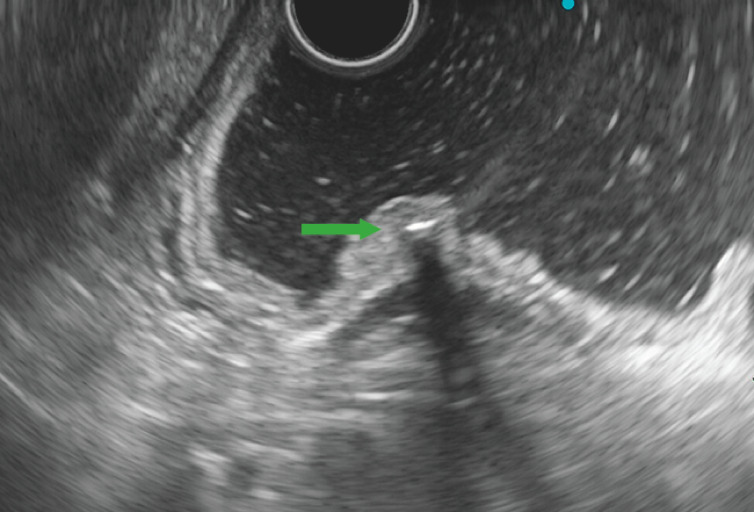
Endoscopic ultrasonography image showing a hyperechoic lesion in the gastric submucosa.

**Fig. 3:**
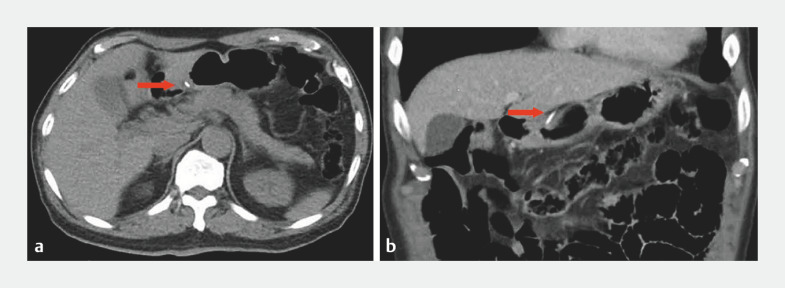
Computed tomography images showing the location and depth of the fishbone (red arrow) on: **a** transverse plane; **b** coronal plane.

Removal of an embedded gastric fishbone by traction-assisted endoscopic full-thickness resection.Video 1

The mucosa of the gastric antrum was circumferentially incised, exposing one side of the fishbone ( Fig. 1 **b** ). Attempts to extract it using foreign body forceps were unsuccessful, indicating significant adhesion with the surrounding tissues ( Fig. 1 **c** ). Snare traction was then employed ( Fig. 1 **d** ). Subsequently, we performed traction-assisted endoscopic full-thickness resection (EFTR), revealing that the base of the fishbone was enveloped within the omentum ( Fig. 1 **e** ). After the adhesions had been dissected, a 2.5-cm long fishbone was successfully extracted ( Fig. 4 ) and the perforation was immediately closed with several metal clips ( Fig. 1 **f** ). The operative and postoperative periods were uneventful, without any complications.

**Fig. 4:**
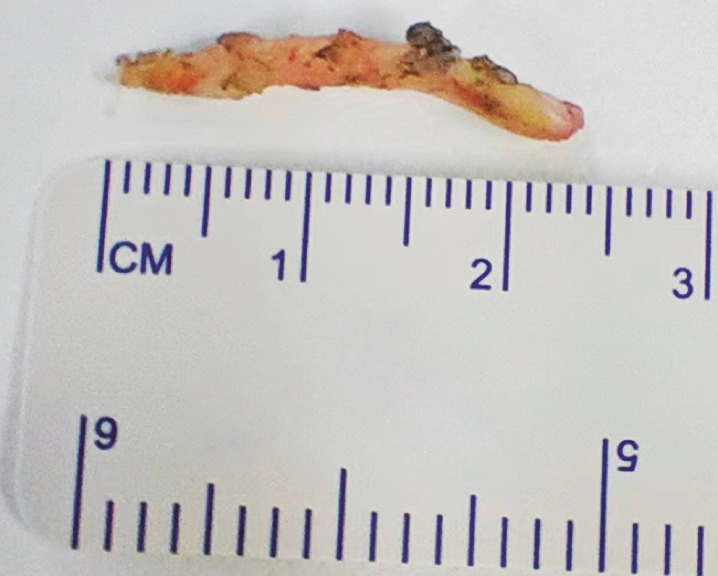
Photograph of the extracted fishbone.

A fishbone invading the intrinsic muscularis and serosa of the gastric wall is rare 1 . Removal is often more challenging when there has been prolonged penetration of the gastric wall, and the risk of complications increases 2 3 . We performed traction using a snare combined with endoclips to assist in ETFR to successfully remove the fishbone. In this case, laparoscopic and open surgery were avoided.

Endoscopy_UCTN_Code_CCL_1AB_2AF

## Notes

Endoscopy E-Videos https://eref.thieme.de/e-videos
*E-Videos* is an open access online section of the journal *Endoscopy* , reporting on interesting cases and new techniques in gastroenterological endoscopy. All papers include a high-quality video and are published with a Creative Commons CC-BY license. Endoscopy E-Videos qualify for HINARI discounts and waivers and eligibility is automatically checked during the submission process. We grant 100% waivers to articles whose corresponding authors are based in Group A countries and 50% waivers to those who are based in Group B countries as classified by Research4Life (see: https://www.research4life.org/access/eligibility/ ). This section has its own submission website at https://mc.manuscriptcentral.com/e-videos .
